# Endolymphatic hydrops imaging and correlation with clinical characteristics, audiovestibular function and mental impairment in patients with Meniere’s disease

**DOI:** 10.1007/s00405-023-07899-w

**Published:** 2023-02-27

**Authors:** Ying Hu, Yue Zhang, Xu Zhao, Juan Li

**Affiliations:** 1 Department of Radiology, Tongji Hospital, Tongji Medical College, Huazhong University of Science and Technology, 1095 Jiefang Avenue, Wuhan, 430030 Hubei People’s Republic of China; 2 Department of Radiology, Wuhan No. 1 Hospital, Zhongshan Avenue #215, Qiaokou District, Wuhan, 430022 Hubei People’s Republic of China

**Keywords:** Meniere’s disease, Endolymphatic hydrops, Vestibular function, Anxiety, Depression

## Abstract

**Purpose:**

MR imaging was used to visualize the vestibular and cochlear endolymphatic hydrops in patients with Meniere’s disease (MD). The relationship between the degree of hydrops and clinical characteristics, audiovestibular function, anxiety and depression state in MD patients.

**Methods:**

70 patients with definitely or probably unilateral Meniere’s disease received bilateral intratympanic gadolinium administration and MR scanning. The degree of bilateral vestibular and cochlea hydrops were analyzed and evaluated by three-dimensional real inversion recovery (3D-real IR) sequence, and the correlation between the grades of endolymphatic hydrops (EH) and disease course, vertigo grading assessment, the duration of vertigo, hearing loss level, caloric test, vestibular myogenic evoked potential (VEMP), electrocochleogram (EcoG), vertigo disability scale (physical, emotional, functional), anxiety and depression scale were studied.

**Results:**

It was found that the vestibule and cochlea EH of the affected and the contralateral ear had different degrees of hydrops and there was no statistical difference between the left and right vestibules. The degree of vestibule EH (V-EH) was significantly positively correlated with the degree of cochlear EH (C-EH). C-EH and hearing loss level were positively correlated with EcoG. There was positive correlation between vestibular EH and hearing loss level, VEMP, caloric test, disease course or vertigo duration. There was a negative relationship between Dizziness Handicap Inventory (Emotion) (DHI(E)) and VEMP. Self-rating Anxiety Scale (SAS) and Self-rating Depression Scale (SDS) scores were positive correlated with DHI(E) and DHI total scores in MD patients.

**Conclusion:**

Endolymph-enhancing MRI was used as an important imaging method for the diagnosis of labyrinthine hydrops in Meniere’s disease. There were certain correlation between EH and the degree of vertigo attack, hearing loss level, vestibular function, and further changes in anxiety and depression emotion.

## Introduction

Meniere’s disease is clinically characterized by recurrent spontaneous attacks of vertigo, progressive or fluctuating hearing loss, tinnitus, aural fullness. The etiology of Meniere’s disease remains controversial. Some scholars believe that the disease may be related to viral infection, autoimmune disease, or endocrine dysfunction. The pathological change of endolymphatic effusion has been confirmed in autopsy [1]. Before the development of various techniques of magnetic resonance imaging (MRI) of lymph in the inner ear, the diagnosis of Meniere’s disease was mainly based on clinical symptoms, pure tone audiometry, caloric test, vestibular evoked myogenic potential, cochlear electrogram and other auxiliary diagnoses. Imaging examination is mainly used to exclude retrocochlear lesions, such as acoustic neuroma. Endolymphatic effusion could not be showed in vivo histopathology. With the further development of MRI of inner ear lymph, many scholars began to carry out a series of studies on whether patients with peripheral vertigo have inner ear lymphatic hydrop and the correlation with various clinical symptoms, hearing and vestibular function tests, in order to explore the further clinical application of MRI on endolymphatic hydrop.

We recruited 70 MD patients who received MRI after diluted gadolinium injected intratympanically, analyzed and evaluated the degree of vestibular and C-EH and studied the correlation between EH and hearing loss level, vestibular function, vertigo symptoms, as well as the anxiety and depression psychological and emotional changes caused by Meniere’s disease.

## Methods

### Participants

We collected 70 patients (70 ears in total) with unilateral definite MD or probable MD diagnosed by the Department of Otolaryngology Head and Neck Surgery in our hospital from 2016 to 2019. The exclusion criteria include: (1) serious cardiovascular diseases or organ failure; (2) serious cervical or lumbar disease, fracture or craniocerebral trauma; (3) middle ear disease, acoustic neuroma, ear trauma, barotrauma, large vestibular aqueduct syndrome or other congenital cochlear malformations. All patients met the diagnostic criteria for Meniere’s disease formulated by BaranySociety [2] in 2015. There were 37 males, 33 females, 42 on the left and 28 on the right, aged 50.8 ± 11.8 years. The clinical data of patients were collected, including disease course, tinnitus, aural fullness, vertigo grading assessment as well as the duration of vertigo (from the onset to the remission time of the patient without drug intervention each time), pure tone audiometry (hearing loss level), caloric test, VEMP, EcoG. All patients completed the questionnaire and filled out the scale of DHI, and SAS, SDS. Basic clinical data and vertigo grading of the 70 patients are presented in Table 1. The study was approved by the Ethics Committee.

**Table 1:** Clinical characteristics and vertigo grading, grading of hearing loss, caloric test, Vemp, EcoG in MD patients

	Meniere’s disease patients
Sex (male/female)	37/33
Age (years)	50.8 ± 11.8
Disease course (years)	0.1–20
Vertigo duration (h)	0.25–6
Tinnitus (positive ratio)	64 (91.4%)
Aural fullness (positive ratio)	47 (67.1%)
Vertigo grade (*n*)	*n* = 70
1	4 (5.7%)
2	13 (18.6%)
3	31 (44.3%)
4	11 (15.7%)
5	9 (12.9)
6	2 (2.9%)
Grading of hearing loss level (*n*)	*n* = 70
0	6 (8.6%)
1	13 (18.6%)
2	11 (15.7%)
3	30 (42.9%)
4	0 (14.3%)
Caloric test (*n*)	*n* = 67
N/CP/CH	29 (43.3%)/28 (41.8%)/10 (14.9%)
VEMP (*n*)	*n* = 31
N/A	21 (52.5%)/18 (47.5%)
EcoG (*n*)	*n* = 64
N/A	21 (32.8%)/43 (67.2%)

*N* normal, *CP* canal paresis, *CH* canal hypofunction, *VEMP* vestibular myogenic evoked potential (VEMP), *EcoG* electrocochleogram

### MRI scanning sequence and parameters

All subjects performed brain MRI to exclude infarction, hemorrhage and tumor lesions. The otorhinolaryngologist conducted gadolinium contrast agent (Gadopentetate Dimeglumine, Magnevist) diluted with eight times normal saline injection through tympanum, respectively [3], keep the injected side ear upward for half an hour, and underwent MRI (Siemens skyra 3 T) 24 h later. The eligible patients were scanned axially with 3D-real reverse version recovery (3D-real IR) sequence. The parameters were as follows: TR = 9000 ms, TE = 179 ms, TI = 1550 ms, layer thickness = 1 mm, layer number = 18, and FOV = 160 mm × 160 mm, Flip angle(FA) = 180 degrees, bandwidth = 213 Hz/Px, matrix = 384 × 384, echo chain length (ETL) = 6, acceleration factor = 2, excitation times = 1, voxel size 0.2 mm × 0.2 mm × 1.0 mm. GRAPPA (parallel imaging technique generalized autocalibrating partially parallel acquisitions) mode was employed, “allowed” was selected in phase partial Fourier and slice partial Fourier of 6/8 to save scan time. Even though, the reduced SNR cannot be detected by our naked eye. Significantly, reconstruction mode was “real” to provide distinct “high signal” in perilymph space. The total scan time was thirteen minutes. Swallowing and coughing should be avoid when scanning was performed. Siemens syngo.via workstation was used for postprocessing including multiplanar reconstruction (MPR) and measurement by two radiology doctors. The area of vestibular endolymphatic and total vestibular were delineated, the degree of cochlear basement membrane displacement and morphology of cochlea duct (endolymphatic fluid) was graded and statistical analyzed.

### EH grading

MR images showed low signal in areas of endolymphatic fluid (black), as opposed to the contrast-enhanced areas in perilymphatic fluid (white) was high signal. The diagnostic criteria refers to Nakashima [4] published in 2009, a simple three-stage grading system was developed for hydrops in both the vestibule and cochlea as follows: none (0), mild (1), and significant (2). In the vestibule, the area ratio of the endolymphatic space to the vestibular space (sum of the endolymphatic and perilymphatic spaces) determined the grading. Values ≤ 1/3 were regarded as no hydrops, values > 1/3 and ≤ 1/2 were regarded as mild hydrops, and values > 1/2 were regarded as significant hydrops. In the cochlea, no displacement of Reissner’s membrane were classified as no hydrops; patients showing displacement of Reissner’s membrane, area of cochlear duct < area of the scala vestibule were classified as having mild hydrops and those area of cochlear exceeding the area of the scala vestibule were classified as having significant hydrops. When EH grading differed between the basal and upper turns of the cochlea, we used the higher EH grading. Siemens syngo.via workstation was used for measurement of the area ratio of the endolymphatic space to the vestibular space. Two radiologists with experience in this field who were blinded to the patients’ diagnoses evaluated the imaging.

### Audiovestibular function tests: PTA, caloric test, VEMP and EcoG

**PTA** The average hearing threshold values of 0.5, 1, 2 and 4 kHz air conductance were taken as the pure tone threshold results. Grading of hearing loss level: grade 1 ≤ 25 dB, grade 2 26–40 dB, grade 3 41–70 dB, grade 4 > 70 dB.

#### Caloric test

The alternate bithermal (50 °C and 24 °C) air caloric test was performed with an interval of 5 min each time and a time of 30 s each time. During the experiment, electronystagmography was used to record nystagmus. Asymmetric ratio (the difference between the bilateral maximum slow velocities/the sum of the bilateral maximum slow velocities) exceeding 22% was an abnormal change. The side with the smaller maximum slow velocity was judged as canal paresis (CP). The side with slow phase velocity (SPV) < 12°was judged as canal hypofunction.

#### Vestibular evoked myogenic potential (VEMP)

The test electrode was placed on the surface skin of the sternocleidomastoid muscle, the reference electrode is placed on the upper part of the sternum, and the forehead is grounded in the middle. During the examination, the patient was instructed to raise his head by 30°, stimulated with a short tone. Distribution recorded bilateral VEMP latency and amplitude, calculated bilateral amplitude ratio and symmetry (IAARs). Bilateral amplitude ratio > 1.61 was abnormal.

#### Electrocochleography (EcoG)

Record the short tone stimulus and short pure tone. The amplitude measurement of SP and AP was based on the starting baseline. When—SP/AP ≥ 0.4, it was judged as abnormal.

### Determination of vertigo degree

#### Grade of vertigo degree self-assessment

According to the diagnostic criteria formulated by AAO-HNS in the United States in 1995 [5], the influence of vertigo on daily life was graded into 1–6 levels (long-term dysfunction). All patients completed questionnaire according to elf description. This functional grading was not only the evaluation of the patients’ current overall functional status, but also the evaluation of the patients' current overall functional status. The data classification and statistics are shown in Table 1

#### Dizziness Handicap Inventory (DHI)

DHI was first published by Jacobson and Newman [6]. It is used to assess the health related quality of life of patients with vestibular system vertigo before and after treatment and self-perception assessment scale, which can assess the severity of subjective symptoms of vertigo patients. The scale consists of 25 questions, including three sub-indexes of Physical(P), Emotion(E), and Function(F). The answers to the questions selected as “yes”, “sometimes” or “no” will be scored 4 points, 2 points and 0 points, respectively, and the final score will be calculated. Grading criteria: 0–30 points, slight obstacle; 31–60: moderate obstacle; 61–100 points: serious obstacle.

#### Self-rating Anxiety Scale (SAS) and Self-Rating Depression Scale (SDS)

SAS and SDS were recommended by the U.S. Department of Education, Health and Welfare for psychopharmacology research, which are filled out by patients in the form of questionnaires. The frequency of symptom occurrence was mainly assessed: “1” means no or little time; “2” means sometimes; “3” means most of the time, and “4” means most or all of the time. Five of the 20 items are positive statements, and 15 items are negative statements. SAS will score them in the order of 1–4 above. The score below 50 points is normal, 50–60 scores is mild anxiety. The score between 61 and 70 is moderate anxiety, and the score above 70 is severe anxiety. Those patients whose scores lower than 50 are normal. Mild depression patients’ scores were 50 or more and less than 60. Score of 60 or more and less than 70 were regarded as moderate to severe depression. Severe depression was defined as a standard score of 70 or more. DHI, SAS, SDS questionnaire statistics are shown in Table 2.

**Table 2:** Descriptive statistics of DHI^a^, SAS^b^, SDS^**c**^ questionnaire

DHI^a^	*n* = 64	SAS	*n* = 67	SDS	*n* = 67
Mild Moderate Serious P E F Total	17 (27.6%) 26 (40.6%) 21(32.8%) 12.8 ± 7.8 13.3 ± 9.4 18.5 ± 10.7 44.6 ± 24.7	None Mild Moderate Serious Total	15 (44.1%) 12 (35.3%) 7 (20.6%) 1 (2.9%) 50.11 ± 11.25	None Mild Moderate Serious Total	16 (47.1%) 10 (29.4%) 5 (14.7%) 3 (8.8%) 49.53 ± 12.81

^a^64 MD patients completed Dizziness Handicap Inventory(DHI) questionnaire

^b^67 MD patients completed Self-rating Anxiety Scale (SAS) questionnaire

^c^67 MD patients completed Self-Rating Depression Scale (SDS) questionnaire

### Statistical analysis

All samples conformed to the normal distribution. The independent sample t-test was used to analyze the difference between the two, and the significance level was set as *p* < 0.05. Spearman rank correlation test for nonparametric variables was used to test the significance of *p* < 0.05.

## Result

### Visualization of endolymphatic hydrops

Seventy patients (ears) were judged for V-EH, 65 patients (ears) were judged for C-EH. Five patients were excluded because of the unclear imaging.

**Fig.1:**
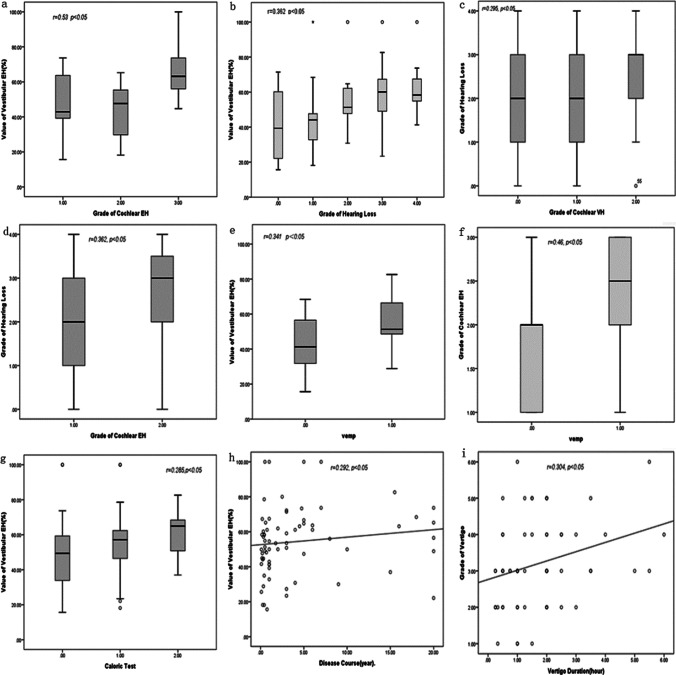
**a** The degree of V-EH was positively correlated with the C-EH (*r* = 0.53, *p* < 0.05). **b** There was a significant correlation between the degree of vestibular hydrops and the hearing loss grade (*r* = 0.362, *p* < 0.05). **c** A significant correlation between the patient with C-EH (all the MD patients) and the hearing loss grade (*r* = 0.295, *p* < 0.05). **d** A more significant correlation between the patient with C-EH (patients without C-EH were exclude) and the hearing loss grade (*r* = 0.362, *p* < 0.05). *x*-axis: 1 = mild C-EH, 2 = significant C-EH. **e** There was a significant correlation between the value of vestibular hydrops EH in MD patients with the VEMP (*r* = 0.341, *p* < 0.05). **f** There was a significant correlation between the cochlear EH in MD patients with the VEMP (*r* = 0.46, *p* < 0.05). **g** There was a significant correlation between the value of vestibular EH and caloric test (*r* = 0.285, *p* < 0.05). *x*-axis: 1 = canal paresis, 2 = canal hypofunction. **h** There was a significant correlation between the value of V-EH and disease course (years) (*r* = 0.292, *p* < 0.05). **i** There was a significant correlation between grade of vertigo of V-EH patients and vertigo duration (h) (*r* = 0.304, *p* < 0.05)

During affected ears, 11 (15.7%) cases had no V-EH, 17 (24.3%) cases had mild V-EH, 42 (60%) cases had significant V-EH. During contralateral ears, 2 of 11 patients without V-EH had vestibular hydrops, 11 of 17 patients with mild V-EH had vestibular hydrops, 12 of 42 patients with significant V-EH had vestibular hydrops. 11 (18.6%) cases had, 23 (32.9%) cases had mild EH in the cochlea, 29 (41.4%) cases had significant EH in the cochlea. in the contralateral cochlea, 1 of 11 case with no EH in the cochlea had mild C-EH, 5 of 23 case with mild C-EH had mild cochlea in contralateral ear, 7 of 29 cases with significant C-EH had significant C-EH. The data classification and statistics are shown in Table 3. The degree of V-EH was positively correlated with the C-EH *r* = 0.53, *p* < 0.01 (Fig. 1).

**Table 3:** Vestibular and cochlear endolymphatic hydrops in MD patients

Grade of EH V-EH C-EH		
	Affected ear: unaffected ear	Affected ear: unaffected ear
None	11 (15.7%): 2	11 (18.6%): 1
Mild	17 (24.3%): 11	23 (32.9%): 5
Significant	42 (60%): 12	29 (41.4%): 7

The correlation of V-EH, C-EH and age disease course, vertigo grading, duration of vertigo, PTA, caloric test, VEMP, EcoG (Table 4, Fig. 1).

1. There was a significant correlation between the value of vestibular hydrops value in MD patients and the hearing loss grade (*r* = 0.362, *p* < 0.05), a more significant correlation between the patient with V-EH (patients without V-EH were exclude) and the hearing loss grade (*r* = 0.464, *p* < 0.01). There was a significant correlation between the degree of cochlear hydrops in all MD patients and the hearing loss grade (*r* = 0.295, *p* < 0.05), a more significant correlation between the patient with C-EH (patients without C-EH were exclude) and the hearing loss grade (*r* = 0.362, *p* < 0.05).
2. There was a significant correlation between cochlear hydrops and EcoG (*r* = 0.271, *p* < 0.05), and between the hearing loss grade and EcoG *r* = 0.342, *p* < 0.05.
3. There was a significant correlation between the value of V-EH and C-EH in MD patients with the VEMP *r* = 0.341, *r* = 0.46, *p* < 0.05.
4. There was a significant correlation between the value of V-EH and caloric test *r* = 0.285, *p* < 0.05.
5. There was a significant correlation between the value of V-EH and disease course (year) *r* = 0.292, *p* < 0.05.
6. There was a significant correlation between grade of vertigo of V-EH patients and vertigo duration(hour) *r* = 0.304, *p* < 0.05.

**Table 4:** Correlation matrix of all variables (Spearman correlation)

	Age	Disease course (years) *n* = 70	Vertigo duration (h) *n* = 70	Grade of vertigo *n* = 70	Grade of hearing loss *n* = 70	Carolic test *n* = 65	EcoG *n* = 64	Vemp *n* = 34	DHI(P) *n* = 64	DHI(E) *n* = 64	DHI(F) *n* = 64	DHI(T) *n* = 64
Value of V-EH	0.09	0.29*	0.05	− 0.05	0.36**	0.29*	0.09	0.34*	0.00	0.00	− 0.02	0.01
Grade of V-EH	0.09	0.23	0.08	0.02	0.46**	0.26*	0.14	0.29	0.11	0.08	0.08	0.11
Grade of C-EH	0.10	0.10	0.22	0.06	0.30*	0.18	0.30*	0.46**	− 0.02	− 0.04	− 0.16	− 0.08
Disease course (years)	0.02		− 0.10	− 0.03	0.22	0.25*	0.07	0.67	0.33**	0.23	0.23	0.30*
Vertigo duration (h)	− 0.07			0.30*	0.04	0.10	0.01	− 0.07	0.05	0.08	0.13	0.11
Grade of vertigo	0.32**				0.14	0.05	− 0.09	0.28	0.09	0.01	0.18	0.14
Grade of hearing loss	0.32**					0.10	0.27*	0.26	− 0.15	0.03	− 0.11	− 0.09
Carolic test	0.06								0.07	0.00	− 0.02	− 0.01
EcoG	0.11								− 0.08	− 0.09	− 0.22	− 0.14
Vemp	0.24								− 0.08	− 0.35*	− 0.17	− 0.24

	SAS value *n* = 28	SAS grade *n* = 28	SDS value *n* = 28	SDS grade *n* = 28	DHI(P) *n* = 28	DHI(E) *n* = 28	DHI(F) *n* = 28	DHI(T) *n* = 28	Disease course (years) *n* = 28	Vertigo duration (h)*’n* = 28	Grade of vertigo *n* = 28	Grade of hearing loss * n* = 28	Vemp
Value of V-EH	0.00	0.06	− 0.05	− 0.01	− 0.18	− 0.29	− 0.40*	− 0.31	0.11	− 0.05	− 0.18	− 0.20	0.13
SAS value					0.21	0.42*	0.37	0.42*	0.11	− 0.10	0.25	− 0.30	0.00
SAS grade					0.08	0.36	0.35	0.33	0.15	− 0.04	0.23	− 0.37	− 0.06
SDS value					0.28	0.388*	0.41*	0.40*	0.22	− 0.04	0.04	− 0.42*	− 0.26
SDS grade					0.20	0.380*	0.40*	0.37	0.24	0.03	0.09	− 0.49**	− 0.43

*p < 0.05 **p < 0.01

### The correlation of mild and significant V-EH and DHI, SAS, SDS (Table 4, Fig. 2)

**Fig.2:**
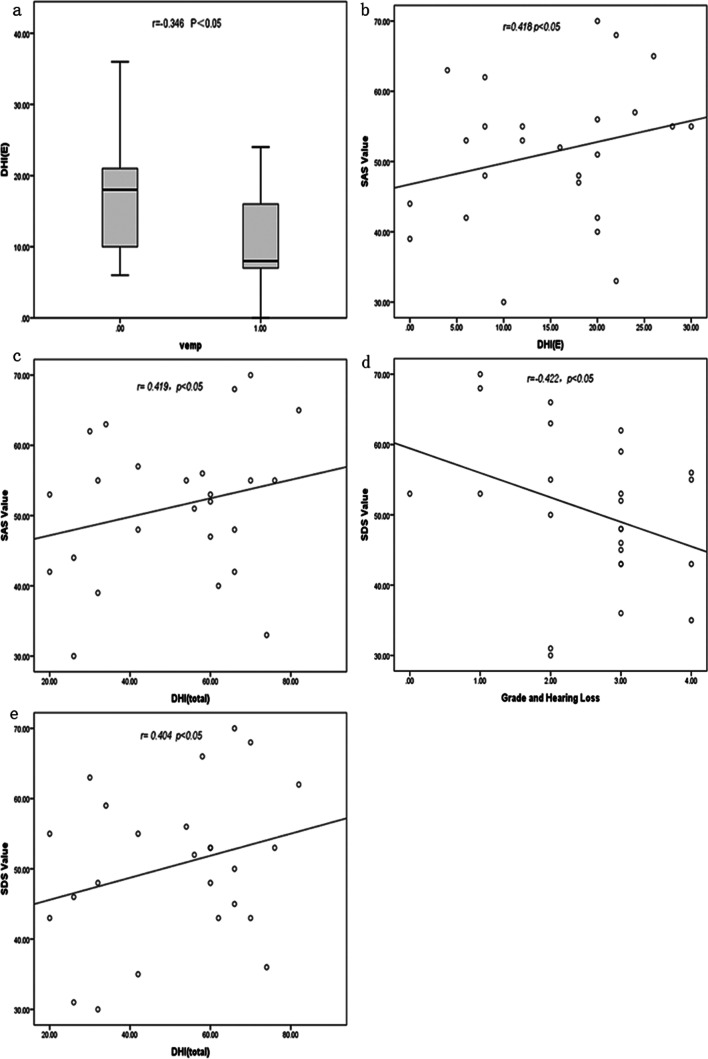
a There was a negative correlation between DHI(E) and VEMP(r = -0.346, p < 0.05) b There was a significant correlation between SAS value and DHI(E) in mild and significant V-EH MD patients (*r* = 0.418, *p* < 0.05). c There was a significant correlation between SAS value and DHI (total) in mild and significant V-EH MD patients (*r* = 0.419, *p* < 0.05). d There was a negative correlation between SDS value and grade of hearing loss in mild and significant V-EH MD patients (*r* = − 0.422, *p* < 0.05). e There was a significant correlation between SDS value and DHI (total) in MD patients (*r* = 0.404, *p* < 0.05)

1. There was a negative correlation between DHI(E) and VEMP *r* = − 0.346, *p* < 0.05.
2. There was a significant correlation between SAS and DHI(E) in MD patients *r* = 0.418, *p* < 0.05. There was a significant correlation between SAS value and DHI (total) *r* = 0.419, *p* < 0.05.
3. There is a difference in hearing loss grade between patients with normal SDS score and patients with abnormal SDS score, *p* < 0.05. There was a negative correlation between SDS value and grade of hearing loss in mild and significant V-EH MD patients *r* = − 0.422, *p* < 0.05.
4. There was a significant correlation between SDS value and DHI (total) in mild and significant V-EH MD patients *r* = 0.404, *p* < 0.05.

## Discussion

MR imaging of endolymphatic hydrops in the inner ear has been investigated by many scholars. Nakashima, a Japanese scholar, used tympanic injection method for the first time to evaluate and quantitatively analyze the hydrops in patients with Meniere’s disease [7]. At present, the scanning sequence mainly include three-dimensional fluid attached inversion recovery (3D-FLAIR) sequence and three-dimensional realversion recovery (3D-real IR) sequence. The latter is more obvious in distinguishing lymph from surrounding bone. In this experiment, we used the method of intratympanic injection through tympanic membrane puncture and 3D-real IR sequence to obtain distinct MRI images.

Li et al. also detected asymptomatic endolymphatic effusion on the non affected side of 32 patients (18%) through MRI of 178 unilateral Meniere’s patients [8]. In addition, some scholars also confirmed the existence of cochlear and vestibular hydrops on the opposite side of unilateral Meniere’s disease patients, and the amount of bilateral hydrops in vestibule is higher than that in cochlea [9]. In our study, 11 of 70 ears of unilateral Meniere’s disease patients had no V-EH, and the most patients had significant hydrops. Some patients had different degrees of V-EH on the lateral ear. It was observed that 13 patients in the contralateral cochlea also had different degrees of C-EH in the patients without or with C-EH, and the incidence of C-EH in the contralateral cochlea was the highest in patients with significant C-EH. In addition, V-EH and C-EH often coexist, showing a significant positive correlation. Consistent with the other researchers, although these patients were diagnosed as unilateral MD clinically, some patients had hydrous changes in the contralateral labyrinth, but did not show corresponding symptoms. Therefore, imaging examination has a positive significance in finding the contralateral inner ear labyrinth changes in patients with unilateral Meniere’s disease. Ratio of the vestibular lymph to total lymph is related to degree of the cochlear hydrops.

Although electrocochleography can help to describe the situation of cochlear hydrops, the fluctuating symptoms of MD limit its clinical application. This study found that the grade of C-EH was positively correlated with the hearing loss level, and the C-EH on the affected side was positively correlated with the EcoG. Patients with mild and significant C-EH showed more obvious positive correlation with hearing loss level, and hearing loss level is related to abnormal EcoG. The results were consistent with those of foreign scholars [10–12]. Hearing loss level plays an important role in the diagnosis of Meniere’s disease. In 1995, the American Association of Otolaryngology, Head and Neck Surgery (AAO-HNS) proposed that MD should be divided into four truncations based on the average pure tone hearing threshold value, rather than vestibular function. However, the diagnostic guidelines for Meniere’s disease formulated by the Bárány Society Guidelines in 2015 recently emphasized the significance of combining the history of attacks of paroxysmal vertigo with low and medium frequency sensorineural hearing loss. Whether the degree of cochlear hydrops can be used as the staging and grading diagnosis of Meniere’s disease in the future remains to be further studied. In addition, we found that cochlear hydrocephalus was related to the duration of the disease.

VEMP is a reflection of saccule function. Some scholars found that the saccule function of patients with EH was significantly lower through correlation analysis of VEMP values and vestibular lymphatic hydrops grades [10]. In our experiment, the percentage of EH in patients with normal and abnormal VEMP was statistically different and correlated (*r* = 0.341, *p* < 0.05), It shows that the EH of MD patients can be used as an index to predict VEMP. It should be pointed out that cVEMP is mainly used in this experimental study, which only reflects the damage of saccule. The presence of EH often causes fluid accumulation in both saccule and utricle, but the saccule and utricle effusion can only be identified in patients with mild and moderate fluid accumulation, while in patients with severe water accumulation, the saccule and utricle effusion are often fused as a whole, which is often difficult to distinguish.

The caloric test reflects the function of the horizontal semicircular canal. In this study, it was found that the caloric test result were related to the degree of the affected ear EH. When the EH was serious, it could extend to the ampulla of the semicircular canal. However, the diameter of the semicircular canal was much smaller. Extremely in severe EH, the ampulla is significantly enlarged, and the perilymphatic space is significantly compressed, so the measurement accuracy is low [13].

The emergence of anxiety in patients with MD has been paid more and more attention by scholars. The symptoms of long-term, recurrent vertigo and fluctuating hearing impairment may be the inducement. The long-term vestibular hyperfunction, inhibition and hearing loss, the variability and unpredictability of vertigo attacks have an impact on the normal life and work of patients, affect their emotions, cause psychological discomfort and even fear. Patients often complain of trance and difficulty in memory and attention. Nausea, vomiting and cold sweat caused by vertigo can cause discomfort in the gastrointestinal system, and chronic instability occurs in the later period of the disease. As early as 1989, scholars have evaluated the psychological status of patients with Meniere’s disease with a scale [14]. Cognitive impairment is common in chronic vestibular diseases such as MD [15] Filipe Correia and other scholars conducted a 5-year horizontal study on MD patients through questionnaires, and found that 80% of patients had dysfunctional personality characteristics, and 34.4% had serious emotional or anxiety disorders [16]. Some scholars found that the diagnosis rate of neurosis and depression (SDS > 40) in patients with refractory MD was significantly higher than that in patients with effective treatment [17]. It can be seen that in addition to improving the therapeutic effect of MD, early psychological intervention is necessary.

In this study, patients diagnosed as mild or significant vestibular and cochlear hydrops were taken as the subjects, the clinical characteristic and the anxiety, depression state and vertigo disability scale were analyzed. Among 70 MD patients, 35.3% were mild and 20.6% were moderate anxiety patients. One patient had severe anxiety, and more than half of all patients had depression. Three of them had severe depression in SDS score. The degree of V-EH patients has a positive correlation with anxiety and depression. The more serious hydrop, the higher level of anxiety, depression or DHI(E) disorder. The more serious EH, the longer the course of the disease, coursed the more obvious the anxiety and depression of MD patients, and the worse the dizziness self feeling of patients reflected by DHI. What was different from previous studies is that in this experiment, it is found that hearing loss is negatively correlated with SDS, but not significantly correlated with SAS. The reason may be related to the sample size, or fluctuating hearing loss of MD patients, or the patient’s stronger tolerance to hearing loss than dizziness, so further research is needed.

Shujian Huang and other scholars found that vestibular dysfunction is related to DHI, and the frequency of vertigo attacks is also related to DHI, DHI and cVEMP were related [18]. Similar to the results of our study, we found that there was a statistical difference in the DHI(E) of patients with normal and abnormal VEMP. There was also a certain correlation between VEMP and the generation of depression, which may be used to predict the change of anxiety and depression in patients.

Our study summarized some characteristics of EH in MD patients. Although the contralateral ear of the unilateral MD patient was asymptomatic, there were varying degrees of EH in vestibular or cochlear. V-EH and C-EH had an impact on the hearing level. VEMP was related to the increase of vestibular hydrops, which might be related to the aggravation of vertigo. Carolic test may be used as a predictor of endolymphatic effusion. Most of the patients with MD have anxiety and depression state, which were related to vertigo, disability and vestibular dysfunction. It is suggested that comprehensive treatment and rehabilitation guidance for MD patients should be strengthened in clinical research, and early intervention and vestibular compensation training should be carried out to provide basis for the recovery of them before and after surgery. So as to improve the prognosis, reduce the impact of anxiety and depression on the quality of life, and break the vicious circle of vestibular symptoms and anxiety.

## Data Availability

The data are available from the corresponding author on reasonable request.
